# Investigation of Health Science Students' Knowledge Regarding Healthy Lifestyle Promotion During the Spread of COVID-19 Pandemic: A Randomized Controlled Trial

**DOI:** 10.3389/fpubh.2021.774678

**Published:** 2021-11-08

**Authors:** Naser Alotaibi, Nowall Al-Sayegh, Mohammed Nadar, Abdulaziz Shayea, Ahmed Allafi, Mohammed Almari

**Affiliations:** ^1^Occupational Therapy Department, Faculty of Allied Health Sciences, Kuwait University, Kuwait, Kuwait; ^2^Department of Physical Therapy, Faculty of Allied Health Sciences, Kuwait University, Kuwait, Kuwait; ^3^Department of Anatomy, College of Medicine, Kuwait University, Kuwait, Kuwait; ^4^Department of Food Science and Nutrition, College of Life Sciences, Kuwait University, Kuwait, Kuwait; ^5^Department of Health Policy and Management, Faculty of Public Health, Kuwait University, Kuwait, Kuwait

**Keywords:** pandemic, physical activity, social media, lifestyle modification, cognition, nutrition, environment, health science curricula

## Abstract

**Background:** Health sciences students as future health care providers, can play a valuable role in protecting societies against the spread of COVID-19 through health promotion and lifestyle modification education. Therefore, proper education of these students is essential.

**Objective:** This study sought to assess and measure the change of knowledge of health sciences students regarding healthy lifestyle promotion strategies during the spread of the Covid-19 pandemic, after participation in different types of online and social media educational programs.

**Methods:** In order to serve the purpose of the study, a methodological research design was first used to ensure the validation of the developed scale; the COVID-19 Healthy Lifestyle Promotion Scale (COVID-19 HLPS). The study utilized a four-arm randomized control research design in which the participants were randomly assigned into one of four groups, (1) control group (placebo intervention), (2) brochure group, who received brochures relevant to healthy lifestyle promotion, (3) Instagram group, who received similar information but through Instagram posts, and (4) online interactive educational workshop group, who also received similar information through an online interactive educational workshop.

**Results:** In total, 155 participants participated in the online and social media intervention programs. There was a significant improvement in the intervention groups in the total knowledge subscale of the healthy promotion strategies compared with the control group (*p* < 0.001). Overall, the workshop group was the most effective group (effect size = 1.54) followed by the Instagram group (effect size = 0.99) and then the brochure group (effect size = 0.91).

**Conclusions:** In order to meet the challenges posed by this pandemic, the use of such online and social media interventions is essential and may be the key for health promotion during this pandemic. Health science students, as future health care professionals, can play a fundamental role during the COVID-19 pandemic in disseminating knowledge relevant to healthy lifestyle to their families and communities thus promoting healthy living and behavioral changes. We propose the development of research initiatives at both national and international levels targeting changes within health science curricula that can meet potential challenges of future pandemics, leading to advancement of health care services globally.

## Introduction

The rapid spread of the novel coronavirus (COVID-19) was not anticipated, and it wreaked havoc worldwide causing unprecedented morbidity and mortality that forced the world to shut down. Strategies to effectively control the spread of the virus were implemented worldwide including full and partial lockdowns and curfews (1). Tools for protecting societies through health promotion and wellbeing during this global pandemic became evident to ensure the control of the outbreak and support the safety of people particularly those at risk (2).

Since the spread of COVID-19 has contributed to the change of people's everyday practices (3), it became essential to consider the key role of health promotion and lifestyle modifications in improving the health and wellness of people's lives through healthy eating habits, promotion of exercise and fitness, the development of structured daily routines and adapting to daily stressors (4). This is more important particularly to the individuals with sedentary lifestyle, or the ones with the existence of comorbidities.

To better control the spread of this pandemic, or address issues pertaining to its spread and effect on people, multidisciplinary health care team members and students are expected to collaborate fully. This in turn contributes to the recovery and minimization of the pandemic severe consequences (5–9). Health science students can play a valuable role in health promotion due the nature of the work they do now, as well as their more critical role as future health care providers. Ideally, they are key workers who need to stay healthy to help others such as family, friends and eventually the public as a whole. Health science students can also serve as a proxy of the general population or the public in facing the spread of this disease. Proper education of these students in this regard is therefore essential.

It has been demonstrated that technology and social media-based interventions can play a significant role in promoting healthy lifestyle among individuals with sedentary lifestyle during this pandemic. Such interventions include the use of mobile health, web-based media (i.e., websites, web pages), wearable devices (i.e., accelerometer) and social media (i.e., Facebook platform) (10). The use of such technology and social media-based interventions were demonstrated to facilitate meaningful teaching experience, and to produce desired learning outcomes for students during the spread of COVID-19 pandemic crisis and beyond (11). In one study where the Instagram platform was utilized among health science students, the study found teaching through Instagram to have positive academic effects and to facilitate academic-student interaction and involvement (12). Another study that employed synchronized online classes among medical student found that the students were in favor of digital teaching and learning, in compassion to traditional teaching, particularly during the COVID-19 lockdown. The study concluded that synchronized online classes are potentially promising for future medical education (13). Having said that, this study aimed to assess and measure the change of knowledge among health sciences students regarding healthy lifestyle promotion strategies during the spread of the Covid-19 pandemic, after participation in different types of online and social media educational programs.

## Methods

### Participants

The study participants included Health Science University students from the disciplines of medicine, pharmacy, physical therapy, occupational therapy, communication disorders, nutrition, medical laboratory sciences, radiological sciences and health informatics and information management. The inclusion criteria were 1- students from health-related disciplines and 2- students who finished the first yr of study in which all general educational requirements were met. The participants who met the inclusion criteria and agreed to participate in the study were randomly assigned into one of four groups, (1) control group (placebo intervention), (2) brochure group, who received brochures relevant to healthy lifestyle promotion, (3) Instagram group, who received the same information provided to the brochure group, but through written text Instagram posts. The Instagram posts did not contain audiovisual or video contents. The last group, and (4) online interactive educational workshop group, received the same information provided to the second and third groups through a synchronous online interactive educational workshop. The sampling was considered based on the following parameters: α (two-tailed) = 0.05, β = 0.2, leading to an 80% power. A minimum sample of 30 participants per group was required to ensure that a real difference between control and intervention groups could truly exist (14).

The process of randomization was performed through an independent biostatistician. The participants were assigned randomly into four groups according to the sample size with the intent of having approximately equal numbers in each group. Randomization was completed using a computer-generated random number sequence. Both the principal researcher and participants were blinded to the allocation.

### Study Design

The study utilized a four-arm randomized control research design to meet the study objective. A methodological research design was also used to ensure the validation of the developed scale; the COVID-19 Healthy Lifestyle Promotion Scale (COVID-19 HLPS).

### Scale Development

An extensive search was conducted using data bases such as Scopus and Medline to identify and generate items relevant to healthy lifestyle promotion specifically pertinent to the COVID-19 pandemic. The COVID-19 Healthy Lifestyle Promotion Scale (HLPS) (Appendix 1) was self-developed by an expert panel of health care professionals (faculty members with a wide range of clinical and teaching experiences: three occupational therapists, two physical therapists, two nutritionists and one public health professional) to assess the perception of the health science students regarding their knowledge about healthy lifestyle promotion strategies during the COVID-19 pandemic. Several meetings were held among the expert panel to ensure having desired items that appropriately address the content of the scale. Several revisions were made to the scale until consensus was reached for the approval of its final version.

The approved final version included 27 items covering several subscales outlined below. A pilot testing phase was then conducted with 39 students from various health science disciplines. The intent of this process was to ensure clarity and relevance of all scale items. To support the content validity of the scale, a content validity index (CVI) was developed. This index included two components; (1). clarity of items (comprehensibility), (2). relevance of items (appropriateness). In addition, for each of these two components, there was a 4-point scale response to choose from for each item (1- strongly disagree, 2- disagree, 3- agree and 4- strongly agree). The magnitudes of floor and ceiling effects were analyzed to support the content validity of the scale. A face validity form was also provided to participants which asked the following question: Do you think that this scale reflected the knowledge base regarding healthy lifestyle promotion pertained to Covid-19 pandemic; the response to this question was dichotomous with either yes or no response. After reviewing and analyzing the results of the content validity index, floor and ceiling effects, and face validity, two questions were deleted, and several items were reworded leaving 25-items for the final scale.

The reliability of the developed scale was assessed in two separate stages. The first stage was conducted after the pilot testing phase where the scale was sent to 90 randomly selected participants. The participants were requested to fill the scale and then after one wk, they were requested to fill it out again. Fifty-two out of the 90 participants completed the scale. The purpose of filling it out twice was to assess the test-retest reliability of the scale. The second stage of measuring reliability was through assessing the internal consistency as well as item-total correlations; this was conducted when the actual data collection process was initiated with a larger sample size.

### Structure and Scoring of the HLPS

**Demographic Data**: Consists of participants' age, gender, email, major, whether or not being educated about the value of other interdisciplinary team member contribution to the health care system, whether or not they learned about the specific role of other disciplines as part of the interdisciplinary team, curriculum coverage of health crisis management within its content, curriculum coverage of the guidelines for infection prevention and control, whether or not they were infected with COVID-19 and whether or not they had an intent to get vaccinated against COVID-19.

**Knowledge Subscale**: Consists of five subscales assessing the knowledge base of the followings:

I. Health and Cognition: 4-items addressing the influence of COVID-19 on cognition (i.e., attention, memory, planning, and problem solving) on cognition, the impact of isolation on the individual's cognition, the impact of COVID-19 on elderly people with cognitive deficits and proper steps to deal with potential cognitive problems due to the COVID-19 pandemic.
II. Daily routine and Environment: 5-items relevant to optimal use of time, proper sleeping patterns, role of spirituality in supporting the individual's mental health, the value of social and virtual environments in promoting health and the importance of public policy in addressing issues relevant to the pandemic.
III. Health and Exercise: 7-items related to types of exercise, recommended minimum guidelines for physical activity as well as factors pertaining to physical fitness contributing to desired health in relation to the spread of COVID-19.
IV. Health and Nutrition: 9-items addressing important information relevant to diet, eating habits and nutritional factors associated with the individual's own illnesses and health to promote a healthy regimen for people that could be at risk of developing COVID-19.
V. Beliefs about Coronavirus Vaccination: 5-items that enquire about the familiarity, effectiveness and duration of immunity relevant to the available vaccines against COVID-19. Of note, this component was added following the pilot testing phase and the test-retest reliability process. However, when assessing the internal consistency and item-to-total correlations with a larger sample size, this component was included. The reason of its addition later on was due to the development and approval of the vaccination afterwards. Hence, the items were developed, reviewed and fully approved by the expert panel prior to its inclusion as part of the scale. Following the addition of this component, the total number of items became 30 items.

The five knowledge subscales consist of a 5-item Likert Scale ranging from 1 (least level of knowledge) to 5 (highest level of knowledge). The total score for each subscale were as follows: health and cognition: 4 questions with a maximum score of 20; daily routine and environment: 5 questions with a maximum score of 25; health and exercise: 7 questions with a maximum score of 35; health and nutrition: 9 questions with a maximum score of 45; and knowledge/beliefs about vaccinations: 5 questions with a maximum score of 25. A higher score indicates better knowledge in healthy lifestyle promotion strategies during the COVID-19 pandemic.

### Procedure

Ethical approval from the local institutional review board was obtained. Students were invited through emails and Microsoft Teams platform to participate in the study. The purpose and procedure of the study was explained to the students and informed consent was obtained. Confidentiality was assured.

Prior to random allocations, the participants were contacted through Microsoft Teams and were asked to fill out the online COVID-19 HLPS. The participants were asked at the end of the scale if they were willing to participate in the intervention programs regarding the acquisition of knowledge relevant to healthy lifestyle promotion during the COVID-19 pandemic. After two wk, all participants who were willing to participate in the intervention programs, and completed one of the four programs, were asked to fill out the COVID-19 HLPS again.

### Statistical Analysis

Descriptive statistics of the data including means, ranges, standard deviations, numbers and percentages were utilized. Internal consistency was measured using Cronbach's alpha coefficient and corrected item-to-total correlations. Test–retest reliability was measured using the intra-class correlation coefficient (ICC). The normality of the data was assessed using the Kolmogorov–Smirnov test. The results of the Kolmogorov–Smirnov test indicated that the data were not normally distributed. Therefore, non-parametric tests were used to analyze the data. Kruskal-Wallis test was used to compare medians of the exposure levels. Probability values (PV) for all multiple comparisons were calculated using Bonferroni adjustment procedure. An alpha *P* < 0.05 was considered statistically significant. For all of the study analyses, the Statistical Package for the Social Sciences (SPSS, version 26) was used.

## Results

The study consisted of 385 participants, with greater participation of women (*n* = 371) than men (*n* = 14). Average age was 20.9 (range = 18 – 35, SD = 2.1). Within their curricula, the majority studied the value of the interdisciplinary approach to the health care system (*n* = 313, 81.3%), while approximately two thirds of the participants specifically studied the key role of these disciplines as part of the interdisciplinary health care team (*n* = 243, 63.1%). Further details of the participant characteristics are outlined in (Table 1).

**Table 1:** Socio-economic and demographic characteristics of the study participants (*N* = 385).

**Characteristics**	***n* (%)**
**Gender**	
Female	371 (96.4)
Male	14 (3.6)
**Age (years)**	
Mean (SD)	20.9 (2.1)
Range	18–35
**Major**	
Medicine	23 (6.0)
Nutrition	14 (3.6)
Pharmacy	14 (3.6)
Allied Health	334 (86.8)
**Has your educational curriculum covered the value of interdisciplinary approach to the health care system?**	
Yes	313 (81.3)
No	72 (18.7)
**Were you educated about the key role of other health care discipline as part of the interdisciplinary team?**	
Yes	243 (63.1)
No	142 (36.9)
**Has your educational curriculum addressed public health crisis management within its content?**	
Yes	184 (47.8)
No	201 (52.2)
**Has your educational curriculum addressed the guidelines for infection prevention and control within its content?**	
Yes	262 (69.1)
No	123 (31.9)
**Have you been infected with COVID-19 before?**	
Yes	54 (14.0)
No	331 (86.0)
**Do you intend to get vaccinated for COVID-19?**	
Yes	250 (64.9)
No	135 (35.1)

**Figure 1:**
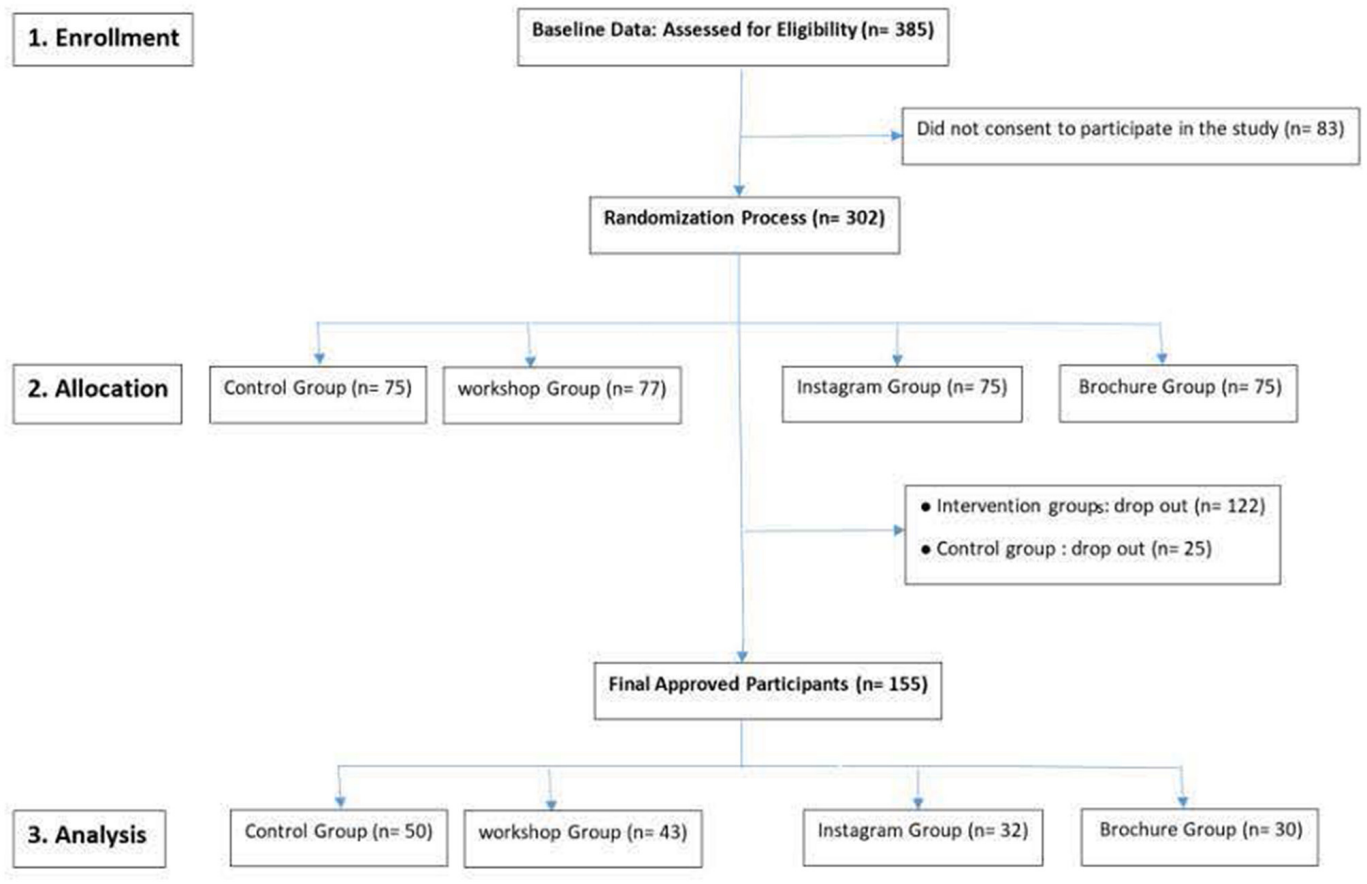
Study flow diagram.

Of the 385 participants, 302 agreed to participate in the online intervention programs and were randomly allocated to the four arms as follows: brochure group (*n* = 75), Instagram group (*n* = 75), online interactive educational workshop (*n* = 77), and control (*n* = 75). During the implementation phase of the online intervention programs relevant to the three intervention groups and control group, 155 participants (51.3%) participated in the online and social media intervention programs: brochure group (*n* = 30), Instagram group (*n* = 32), online interactive educational workshop (*n* = 43), and control group (*n* = 50); specific details of the enrollment, allocation and analysis are illustrated in the study flow diagram (Figure 1).

With regards to the reliability of the scale, Cronbach's α-value for the knowledge scale was 0.909 and the Item-to-total correlations ranged from 0.289 to 0.640 (*n* = 385) indicating a satisfactory internal consistency as well as Pearson's correlation coefficients. In addition, the ICC of the scale was 0.896 (95% CI 0.818–0.940) (*n* = 52) suggesting an excellent agreement. Further description of the reliability of the scale is provided in (Table 2).

**Table 2:** Reliability analysis of the HLPS and its subscales (*N* = 385).

**Subscale**	**Cronbach's**	**Item-to-total**	**ICC^b^**

	**Alpha^a^**	**Correlations**	**(95% CI)**
**Knowledge (30-items)^c^**	0.909	0.289–0.640	0.896 (0.818–0.940)
**Health and Cognition (4-items)**	0.744	0.489–0.583	0.784 (0.625–0.876)
**Lifestyle and Environment (5-items)**	0.724	0.427–0.539	0.722 (0.515–0.841)
**Health and Exercise (7-items)**	0.856	0.422–0.730	0.867 (0.769–0.924)
**Health and Nutrition (9-items)**	0.855	0.475–0.697	0.801 (0.652–0.886)
**Vaccination Knowledge & Beliefs (5-items)**	0.787	0.483–0.616	-

a *Cronbach's alpha was based on N = 385*.

b *ICC was based on N = 52*.

c *Knowledge subscale ICC is calculated based on four subscales (Fifth component was added later)*.

**Figure 2:**
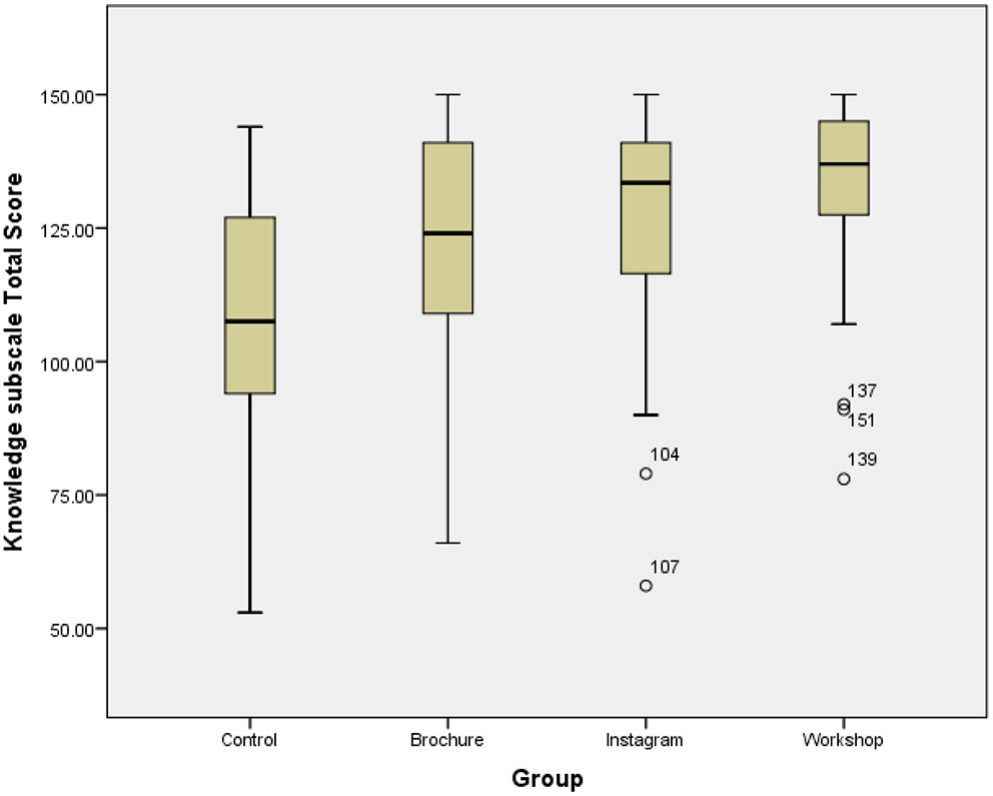
Comparison boxplot of the total knowledge subscale score for the control group and the intervention groups.

Table 3 describes participant knowledge within groups of the different subscales. Overall, the workshop group was the most effective group (effect size = 1.54) followed by the Instagram group (effect size = 0.99) and then the brochure group (effect size = 0.91). As shown in (Table 4), there was a significant improvement in the intervention groups in the total knowledge subscale of the healthy promotion strategies compared with the control group (*p* < 0.001). Total knowledge subscale score for the control group and intervention groups are illustrated in the boxplot (Figure 2).

**Table 3:** Participant's knowledge subscale Pre-post (within group).

**Subscale**	**Control**		**Brochure**		**Instagram**		**Workshop**	
**component**	**(***n*** **=** **50)****		**(***n*** **=** **30)****		**(***n*** **=** **32)****		**(***n*** **=** **43)****	
	**Post**	**Pre**	**Post**	**Pre**	**Post**	**Pre**	**Post**	**Pre**
**Health and cognition**								
Mean (SD)	13.0 (3.5)	10.72 (3.94)	16.0 (3.0)	11.9 (4.5)	15.5 (3.4)	11.1 (4.0)	16.9 (2.9)	10.9 (4.0)
Range	4–20	4–20	11–20	4–20	8–20	4–20	8–20	4–20
*P* Value^*^	0.002		0.001		0.001		0.0001	
Effect size	0.58		0.91		1.1		1.5	
**Lifestyle and environment**								
Mean (SD)	18.2 (4.1)	18.5 (3.7)	20.5 (3.7)	18.8 (4.3)	22.1 (3.0)	19.2 (4.4)	22.2 (3.6)	18.6 (4.2)
Range	9–25	12–25	13–25	5–25	14–25	6–25	12–25	9–25
*P* Value^*^	0.715		0.087		0.008		0.001	
Effect size	0.08		0.40		0.66		0.86	
**Health and exercise**								
Mean (SD)	25.1 (6.7)	23.3 (6.8)	28.4 (5.0)	23.4 (6.8)	28.8 (7.0)	23.2 (7.2)	31.2 (4.2)	22.6 (6.7)
Range	7–35	8 – 35	14–35	7–35	9–35	7–35	17–35	9–35
*P* Value^*^	0.209		0.005		0.008		0.0001	
Effect size	0.26		0.74		0.78		1.28	
**Health and nutrition**								
Mean (SD)	35.6 (8.2)	33.4 (8.3)	37.3 (7.2)	33.4 (7.3)	38.6 (7.4)	35.9 (7.8)	40.7 (5.4)	33.7 (7.4)
Range	10–45	9 – 45	18–45	10–45	17–45	18–45	25–45	18–45
*P* Value^*^	0.759		0.046		0.135		0.0001	
Effect size	0.27		0.53		0.35		0.95	
**Vaccination knowledge & beliefs**								
Mean (SD)	17.2 (4.3)	14.0 (5.3)	20.5 (4.1)	14.6 (5.2)	21.1 (4.1)	14.7 (5.2)	22.5 (3.8)	14.1 (5.7)
Range	8–24	5–25	9–25	5–25	10–25	6–25	8–25	5–25
*P* Value^*^	0.004		0.001		0.001		0.0001	
Effect size	0.60		1.13		1.23		1.47	
**Knowledge subscale total score**								
Mean (SD)	107.1 (22.8)	99.9 (20.5)	122.7 (20.5)	102.1 (22.6)	126.1 (22.4)	104.1 (22.3)	133.4 (16.8)	99.9 (21.7)
Range	53–144	45–135	66–150	31–150	58–150	46–149	78–150	56–146
*p*-value^*^	0.156		0.002		0.001		0.0001	
Effect size	0.35		0.91		0.99		1.54	

* *P Value was calculated using Wilcoxon signed rank test on the differences due to pairing*.

**Table 4:** Participant's knowledge subscale (between group).

**Subscale component**	**Control**	**Brochure**	**Instagram**	**Workshop**	***P* value**
**Health and cognition**					
Mean (SD)	13.0 (3.5)	16.0 (3.0)	15.5 (3.5)	16.9 (2.9)	<0.001
**Lifestyle and environment**					
Mean (SD)	18.2 (4.1)	20.5 (3.7)	22.1 (3.0)	22.2 (3.6)	<0.001
**Health and exercise**					
Mean (SD)	25.1 (6.7)	28.4 (5.0)	31.2 (4.3)	29.8 (4.2)	<0.001
**Health and nutrition**					
Mean (SD)	33.6 (8.2)	37.3 (7.2)	38.6 (7.4)	40.7 (5.4)	<0.001
**Vaccination knowledge & beliefs**					
Mean (SD)	17.2 (4.3)	20.5 (4.1)	21.1 (4.1)	22.5 (3.8)	<0.001
**Knowledge subscale total score**					
Mean (SD)	107.1 (22.8)	122.7 (20.5)	126.1 (22.4)	133.4 (16.7)	<0.001

* *P Values were calculated using Kruskal-Wallis test comparing equality of medians since the scores in at least one group were not normally distributed*.

## Discussion

This study investigated the knowledge of health sciences students regarding healthy lifestyle promotion strategies during the spread of the COVID-19 pandemic after their participation in different educational programs. The results found significant improvements in all of the intervention groups compared to the control group with the online interactive educational workshop group followed by the Instagram group consistently outperforming the other groups. In addition, the findings support the use of an online platform and social media-based interventions, particularly during a pandemic in promoting healthy lifestyle knowledge among various health care students. Our finding complement previous studies which demonstrated the value and effectiveness of virtual education, such as online and social media intervention programs, for students in their academic environment (11–13).

Such interventions are also supported by the literature with other populations than students, thereby contributing to the learning and intellectual exchange as well as provisions of health care services, such as maintaining the expected social distancing among people (10, 15). For example, it was reported that social media-based interventions appear to be promising methods for promoting health and wellbeing, especially during the pandemic. Therefore, the need for alternative means of population-based interventions that alter the ways in which society changes their lifestyle and habits may be addressed with such virtual strategies. As a result, health professionals who engage in healthy lifestyle behaviors are more likely to promote healthy living in their communities and educational programs that emphasize healthy diet, exercise, and stress management (16). Therefore, online and social media interventions including online interactive educational workshops, Instagram posts and brochures, can be vital for health promotion during this pandemic. Thus, starting with health care professionals or future health care professionals is a step in the right direction. The risks associated with sedentary lifestyle during lockdowns or partial curfews may be overcome through the social support that these platforms and social medias provide in promoting healthy living and behavioral changes.

The virtual environment can serve as a key factor to provide knowledge as an important source of support to people, particularly due to lockdown and possibly limited socialization. For example, the use of virtual environment, such as electronic and social media as well as web-based communication tools, can be a substitute to facilitate communication, connectedness and learning opportunities thus mitigating the challenges imposed by this pandemic (17–20). Furthermore, it is of importance to note that the use of virtual learning and technology can also be applied cross-culturally to promote accessible learning and guide an international collaborative health education around the world (12). Doing so can contribute to international research opportunities among various University academicians as well as health care professionals to support optimum health care services.

About two thirds of the participants were educated about the specific role of other health care disciplines as part of the interdisciplinary team, which raises a concern that might influence the provision of desired service delivery. Hence, curricular changes of integrating more focus on advancing students' knowledge base about other disciplines domains and practice can assist and prepare students, as future health care professionals, for challenges imposed by the pandemic or potential future pandemics (21). Understanding of how these disciplines are interlinked can also lead to better preparation and adaptation to meet such unprecedented crises in the future. In addition, less than half of the study participants (47.8%) reported that their educational curriculum addressed public health crisis management which further requires changes to be made. It is recommended therefore for health care departments to consider modifications of their curricula to meet the new demand of such global change resulting from newly developed pandemics leading to proper epidemic control in the future (22, 23).

Moreover, addressing the guideline for infection prevention and control within the education curriculum, as reported by the study participants, was about 70% which needs to be even more embedded within its content. The inclusion of the comprehensive coverage of guidelines for infection prevention and control within the health science educational curricula is highly encouraged. This is a vital step that can aid in the management of viral pandemic outbreaks possibly encountered in the future (24). For example, preparing health care students, such as medical students, to tackle problems or issues relevant to the pandemic can serve as a fundamental component required to be part of their educational curricula (25). Having said that, we propose the development of research initiatives both at the national and international levels targeting changes within health science curricula that can meet all potential challenges of future pandemics. In turn, this will guarantee adequate level of preparedness towardz any viral or infection threat encountered leading to improvement and advancement of health care services globally. Nonetheless, policy making, and governmental legislations are crucial elements to ensure prevention as well as control of the COVID-19 Pandemic in their own countries. Thus, investments in health resources and funding, taking desired precautionary measures and choosing appropriate response strategies are all essential solutions to control and minimize the spread and effect of this pandemic globally (26, 27).

The strengths of the study are of significant value and should be indicated. First, to our knowledge, this study is the first of its kind to examine the use of online platforms and social media interventions, to acquire knowledge relevant to healthy lifestyle promotion strategies during the COVID-19 pandemic, with health science students of different disciplines using a rigorous research design. Second, this study supports the holistic view of interdisciplinary teamwork and collaboration, while addressing individuals' healthy lifestyle promotion strategies reflecting various domains including cognition, daily routine and environment, exercise, nutrition and knowledge and beliefs about vaccinations. Such collaboration is highly encouraged whether in the areas of assessment or intervention to facilitate fruitful research-related opportunities. Third, the study findings encourage health care educators to support the use of online interactive workshops, Instagram posts and brochures as teaching strategies within their curricula to promote students' learning and educational outcomes. Fourth, the psychometric properties established in this study for the COVID-19 Healthy Lifestyle Promotion Scale (COVID-19 HLPS) allow future studies to use this outcome measure to document, monitor and track wellness programs targeting healthy lifestyle promotion of different populations.

### Limitations

The first limitation is that the percentages of students from the different majors were not equally represented. Second, the findings relevant to the curriculum contents may not have been comprehensive as the years of study were not reported. Third, the observed gap in educational curriculum regarding public health crisis management was made from a small sample, and from a single university. Future studies should therefore utilize large-scale comparisons to verify whether this gap is frequent. Another limitation of this study is that we did not measure the students' generalizability of the knowledge gained in their environment during the pandemic. We encourage future studies to pay more attention to external validity and enhance generalizability to facilitate more appropriate use of research findings. We further recommend indicating the specific year of study for the students to allow in-depth understanding of the curriculum content throughout the years of study.

## Conclusion

During the spread of the Covid-19 pandemic, the use of online platforms and social media interventions including online interactive educational workshops, Instagram posts and brochures are of great value to students' knowledge relevant to healthy lifestyle promotion strategies. Therefore, to meet the challenges posed by this pandemic, the use of such online and social media interventions is essential and may be key for health promotion during pandemics. Health science students, as future health care professionals, can play a fundamental role during the COVID-19 pandemic in disseminating knowledge relevant to healthy lifestyle to their families and communities thus promoting healthy living and behavioral changes. In addition, the study findings can encourage health care faculty members to support the use of online educational interactive workshops, Instagram posts and brochures as teaching strategies within their curricula to promote students' learning and educational outcomes. Furthermore, future studies can use the COVID-19 Healthy Lifestyle Promotion Scale (COVID-19 HLPS) as a standardized outcome measure to document, monitor and track wellness programs evaluation targeting healthy lifestyle promotion of different populations. Finally, we propose the development of research initiatives at both national and international levels targeting changes within health science curricula that can meet potential challenges of future pandemics, leading to improvement and advancement of health care services globally.

## Data Availability Statement

The original contributions presented in the study are included in the article/Supplementary Material, further inquiries can be directed to the corresponding author.

## Ethics Statement

The studies involving human participants were reviewed and approved by Institution Review Board (IRB) at Kuwait University (VDR/EC/36/8). The participants provided their written informed consent to participate in this study.

## Author Contributions

NaA, NoA, MN, AS, AA, and MA: conceptualization, methodology, and resources. NaA, NoA, and MN: validation. NaA and NoA: formal analysis, writing—original draft preparation, funding acquisition, and supervision. NaA, NoA, and MN: investigation and writing—review and editing. NaA, NoA, MN, and AS: data curation and project administration. All authors have read and agreed to the published version of the manuscript.

## Funding

The project was funded by Kuwait Foundation for the Advancement of Sciences under project code: PN20-13NO-02.

## Conflict of Interest

The authors declare that the research was conducted in the absence of any commercial or financial relationships that could be construed as a potential conflict of interest.

## Publisher's Note

All claims expressed in this article are solely those of the authors and do not necessarily represent those of their affiliated organizations, or those of the publisher, the editors and the reviewers. Any product that may be evaluated in this article, or claim that may be made by its manufacturer, is not guaranteed or endorsed by the publisher.

## Supplementary Material

Appendix 1 COVID-19 healthy lifestyle promotion scale (COVID-19 HLPS).
