# Supplementary material for: Investigation of Health Science Students' Knowledge Regarding Healthy Lifestyle Promotion During the Spread of COVID-19 Pandemic: A Randomized Controlled Trial
Source: Front Public Health. 2021 Nov 8;9:774678. doi: 10.3389/fpubh.2021.774678 (PMC8606582; doi:10.3389/fpubh.2021.774678)
Supplement: Appendix 1 — COVID-19 healthy lifestyle promotion scale (COVID-19 HLPS). [file Data_Sheet_1.pdf]

Appendix 1: COVID-19 Healthy Lifestyle Promotion Scale (COVID-19 HLPS)

First Section: Demographic Data

1-Age:

2-Gender: - Male - Female

3- Email:

4-Major: -1- Medicine, 2- Pharmacy, 3-Physical Therapy, 4-Occupational Therapy,

5- Medical Laboratory Sciences, 6-Radiological Sciences, 7- Health Informatics and Information Management, 8- Nutrition, 9- Communication Disorders

5-Has your educational curriculum covered the value of interdisciplinary approach to the

healthcare system:

-Yes -No

6- Were you educated about the key role of other health care discipline as part of the interdisciplinary team:

-Yes -No

7-Has your educational curriculum addressed public health crisis management within its content:

-Yes -No

8- Has your educational curriculum addressed the guidelines for infection prevention and control within its content:

-Yes -No

9. Have you been infected with COVID-19 before:

-Yes -No

10. Do you intend to get vaccinated for COVID-19:

-Yes -No

Second Section: Knowledge Subscale

First Component: Health and Cognition					
	**Least knowledgeable**				**Most knowledgeable**
1. I am aware about the influence of COVID-19 on cognition (eg. Attention, memory, planning, problem solving)	1	2	3	4	5
2. I am aware that people with dementia, mild cognitive impairment, or mental disorders are more vulnerable to cognitive deterioration after social isolation	1	2	3	4	5
3. I am aware that people with dementia are more susceptible to develop delirium (a state of mental confusion and emotional disruption) if they develop an infection, such as COVID-19	1	2	3	4	5
4. I know the proper steps to deal with potential cognitive problems an individual might face after a COVID-19 infection	1	2	3	4	5
**Second Component: Daily Routine and Environment**					
	**Least knowledgeable**				**Most knowledgeable**
5. I know the best intervention strategies to support the optimal use of my time during the present COVID-19 pandemic	1	2	3	4	5
6. I understand the best strategies to address issues related to sleep disturbances or lack of proper sleeping pattern during the present COVID-19 pandemic	1	2	3	4	5
7. I understand the role of spirituality in improving the mental health and wellbeing of individuals during the present COVID-19 pandemic	1	2	3	4	5
8. I understand the key role of social environment as well as virtual environment (i.e. internet and social media) in promoting health for the individuals and communities during the present COVID-19 pandemic	1	2	3	4	5
9. I understand how public policy can affect the individual and community with relevance to issues targeting the management of the present COVID-19 pandemic	1	2	3	4	5
**Third Component: Health and Exercise**					
	**Least knowledgeable**				**Most knowledgeable**
10. I am familiar with safe and effective types of exercises to help maintain and/or improve my health while reducing the risk of the spread of COVID-19	1	2	3	4	5
11. I am aware about the types of exercises that contribute to reducing the risks of developing chronic non-communicable diseases (eg. Diabetes, hypertension) that are associated with severe adverse effects from COVID-19	1	2	3	4	5
12. I am familiar with the current recommended minimum guidelines for physical activity to stay fit and healthy especially during the COVID-19 pandemic	1	2	3	4	5

13. I am familiar with potential perceived barriers that prevent individuals from being moderately physically active during the COVID-19 pandemic	1	2	3	4	5
14. I am familiar with how to set goals and monitor outcomes of exercise programs while considering how to minimize the risk of COVID-19	1	2	3	4	5
15. I am aware of how to build social support to keep physically active while minimizing the risk of COVID-19	1	2	3	4	5
16. I am familiar with the negative effects of sitting for prolonged periods of time on one's health, especially as it relates to COVID-19	1	2	3	4	5
**Fourth Component: Health and Nutrition**					
	**Least knowledgeable**				**Most knowledgeable**
17. I understand that eating the same foods everyday may affect my health and wellbeing	1	2	3	4	5
18. I am aware of the relationship between healthy fats intake and lowered immunity and inflammation	1	2	3	4	5
19. I am familiar with the sources of Trans fat in my diet	1	2	3	4	5
20. I am familiar with the fiber content of foods and how that impacts my gut health	1	2	3	4	5
21. I understand the influence of stress on my eating habits and how it may affect controlling my body weight	1	2	3	4	5
22. I am aware about the dietary recommendations and their importance on my health	1	2	3	4	5
23. I am familiar with the effect of sugar, salts, and fats on appetite control	1	2	3	4	5
24. I understand the importance of practicing good food hygiene to minimize food-borne illnesses and infections	1	2	3	4	5
25. I understand how unhealthy diet is associated with various diseases	1	2	3	4	5
**Fifth Component: Knowledge and beliefs about coronavirus Vaccination**					
	**Least knowledgeable**				**Most knowledgeable**
26. I know how effective is the coronavirus vaccine	1	2	3	4	5
27. I am familiar of the side effects of the coronavirus vaccine	1	2	3	4	5
28. I know that someone who was vaccinated for coronavirus can still spread the virus	1	2	3	4	5
29. I understand the difference between the different coronavirus vaccines (Pfizer, Moderna, Oxford,..)	1	2	3	4	5
30. I am aware of the durations of immunity after receiving the coronavirus vaccine	1	2	3	4	5
